# Improved Faster R-CNN Traffic Sign Detection Based on a Second Region of Interest and Highly Possible Regions Proposal Network

**DOI:** 10.3390/s19102288

**Published:** 2019-05-17

**Authors:** Faming Shao, Xinqing Wang, Fanjie Meng, Jingwei Zhu, Dong Wang, Juying Dai

**Affiliations:** Department of Mechanical Engineering, College of Field Engineering and Army Engineering University, Nanjing 210007, China; shaofaming@163.com (F.S.); Beilimeng1992@163.com (F.M.); zhu_jingwei@sohu.com (J.Z.); dyhkxydfbb@163.com (D.W.); dinajy2001@126.com (J.D.)

**Keywords:** simplified Gabor filters, Faster R-CNN, secondary regions of interest, highly possible regions proposal

## Abstract

Traffic sign detection systems provide important road control information for unmanned driving systems or auxiliary driving. In this paper, the Faster region with a convolutional neural network (R-CNN) for traffic sign detection in real traffic situations has been systematically improved. First, a first step region proposal algorithm based on simplified Gabor wavelets (SGWs) and maximally stable extremal regions (MSERs) is proposed. In this way, the region proposal a priori information is obtained and will be used for improving the Faster R-CNN. This part of our method is named as the highly possible regions proposal network (HP-RPN). Second, in order to solve the problem that the Faster R-CNN cannot effectively detect small targets, a method that combines the features of the third, fourth, and fifth layers of VGG16 to enrich the features of small targets is proposed. Third, the secondary region of interest method to enhance the feature of detection objects and improve the classification capability of the Faster R-CNN is proposed. Finally, a method of merging the German traffic sign detection benchmark (GTSDB) and Chinese traffic sign dataset (CTSD) databases into one larger database to increase the number of database samples is proposed. Experimental results show that our method improves the detection performance, especially for small targets.

## 1. Introduction

Traffic sign detection and recognition has attracted increasing attention in recent years. This has not only included in-depth academic research, but also has wide application in commercial aspects. Well-known car companies, such as BMW and Mercedes-Benz, are actively investing in road environmental perception system (REPS) research. The REPS systems not only include front vehicle detection and pedestrian detection, but also traffic sign detection systems for warning drivers to pay attention to traffic signs around the road. Many traffic accidents occur because pedestrians or drivers do not notice traffic signs. With the increasing number of vehicles, the increasing pressure of traffic safety and the demand for intelligent vehicle development, it is necessary to use computer technology to automatically detect and recognize traffic signs. Research in this area has been underway since the 1980s.

In general, the design of artificially constructed objects—for example, car design may focus on speed, beauty, and safety; chair design may focus on comfort, etc.—does not consider easy recognition. In contrast to tanks and military vehicles, for which camouflage is often applied to make them difficult to detect, traffic sign design pays great attention to recognition attributes. In order to attract attention, traffic signs are often designed with strict color and shape and contain special symbols that make them easy to detect from traffic scenes. The features mentioned above are also the key information for traffic sign detection research. However, the shape, color, and symbolic information of traffic signs will not always be stable in an actual road environment because there are many adverse factors, such as viewpoint variations, physical damages, bad weather, etc. This makes the detection and recognition of traffic signs challenging for both humans and computer systems. The difficulties faced include the following:

- Traffic scene images are often subject to motion blur because the images are captured from the camera of a vehicle traveling at a high speed.
- Since vehicle-mounted cameras are not always perpendicular to traffic signs, the shapes of traffic signs are usually distorted in captured images, and the shape of traffic signs in traffic scenes are not always be reliable.
- Some traffic signs are often obscured by other objects in the road, such as trees, pedestrians, other vehicles and so on. Therefore, it is necessary to detect traffic signs with only part of the traffic sign image information.
- The problems of traffic sign discoloration, traffic sign damage, rain, snow, fog, and other factors also produced enormous difficulties in traffic sign detection.

**Figure 1:**
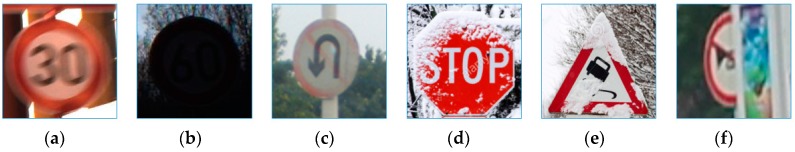
Examples of the unideal traffic sign images: (**a**) motion blur, (**b**) undesirable light, (**c**) color fading, (**d**) and (**e**) snow, (**f**) occlusion.

Some challenging examples are shown in Figure 1.

In this paper, a real-time traffic sign detection system and a super class classification method are presented. This method results in comparable detection and classification accuracy with state-of-the-art methods. The main contributions of the paper are outlined as the following:

- A highly possible regions proposal network (HP-RPN) for regions filtering is presented, which provides important regional proposal reference information to a modified Faster R-CNN, and filters out most non-traffic sign areas.
- In order to solve the problem of less feature information from small targets in the fifth layer of the VGG16 network, the features of its third, fourth, and fifth layers are fused; which greatly improves the feature expression ability for small target detection.
- The secondary region of interest (SROI) is proposed to introduce structural information other than traffic signs into the detection network, which further improves the detection efficiency.

The rest of this paper is organized as follows: Section 2 introduces the related work. Section 3 gives an overview of our methods. The details of the traffic sign detection method are presented in Section 4. Section 5 discusses the experimental evaluation. Finally, some conclusions and identified future work are presented.

## 2. Related Work

Traffic sign detection has been increasingly studied as an important branch of object detection. Traffic sign detection has certain similarities with general object detection, such as face detection [1,2,3], iris detection [4], and small object detection on remote sensing images [5]. However, traffic sign detection has its own particularities. Compared with iris and medical image detection, the artificial design features of traffic signs are more obvious. The scale and proportion of traffic signs in the detected image are also very different. Furthermore, the number of classes of traffic signs and the size of the required training database makes it impossible for us to directly apply the other object detection methods mentioned above to traffic sign detection. Researchers need to make appropriate amendments and improvements according to this specific situation.

### 2.1. Detection Framework

Traffic sign detection belongs to the domain of target detection. The object detection framework determines the detection process. The main objective of this framework optimization is to avoid missing detection and redundant detection. Therefore, the framework structure of object detection often determines the detection speed. For real-time object detection, the detection framework is very important. The current popular target detection frameworks generally include region-based target detection and proposal-free methods. Region-based target detection first generates thousands or hundreds of proposal regions in the input image. These two-dimensional regions are then transformed into fixed-length one-dimensional vectors, which are then classified by classifiers and adjusted by their position. The pioneering work of region-based target detection began with R-CNN [6], which includes three modules: region proposal, vector transformation and classification. SSP-net [7] optimizes R-CNN in many aspects and improves the detection performance. Fast R-CNN [8] integrates the essence of R-CNN and SPP-net and introduces a multitask loss function, which makes the training and testing of the whole network very convenient. Faster R-CNN [9] uses the RPN to replace the selective search module in Fast R-CNN and RPN shares features with the Fast R-CNN, this improvement greatly improves both the time and accuracy of target detection. 

Another type of target detection framework that does not require a region proposal process which is called proposal-free approach. The OverFeat method [10] classifies detection regions by sliding windows with different scales at each feature point on the topmost feature layer. YOLO [11] classifies and locates the objects in one step. YOLO directly regresses the location of the bounding box and the category of the bounding box in the output layer, thus realizing one-stage. SSD [12] uses convolution kernels on feature maps to predict the class and coordinate offsets of a series of default bounding boxes. Compared with the region based methods, the proposal-free methods also have better detection accuracy and speed. 

### 2.2. Feature Expression

The accuracy of detection and classification often depends on the feature expression of the detection object. The feature expression of the detection object includes two aspects: one is the feature expression of the region to which the detection object belongs; the other is the feature expression outside the detection region, which involves the fusion of the context information. Small target detection has some problems such as blurred images, low information and being easy to misjudge. So the feature expression of small target detection is particularly important.

Because the RGB color space is unstable and unreliable, the method of improving the detection efficiency by transforming the original image features has been widely used in the initial stage of object detection, which is called a hand-crafted feature [13,14,15]. However, the hand-crafted feature belongs to the low-level features, and the data dimension is large, so it does not have the high-level semantic expression ability, the improvement effect is not obvious and the improvement space is small. Compared to traditional hand-crafted features, the application of deep features in the field of target detection has achieved better and better detection results. The R-CNN method [6] used the trained CNNs to classify the target area and then judge whether it belongs to the target or the background. The Faster R-CNN [9] used the fifth layer of VGG16 as the detection and classification feature which gets state-of-the-art results. 

Compared with using only one layer of deep convolution features, more and more methods use feature fusion methods such as multi-layer, multi-scales, etc. The deep convolution feature was used to achieve richer feature expression [16,17,18,19]. Ren et al. [5] use three layers of deep features in ResNet-50 to detect small objects in optical remote sensing images. In Li and Yu [16], in order to detect objects, multi-scales features from deep CNNs were used to detect the saliency map for different levers of image segmentation. Hou et al. [17] used the information of multi-scale and multi-level deep features of fully convolutional neural networks which provide more semantic information. Therefore, the application of deep feature information or deep feature fusion in target detection is indeed a noteworthy research direction. 

As many methods have focused solely on the detection target itself [9,11,12,20,21], the relationship between the detection target and the environment in which the target is located has often been neglected. This information is usually conducive to the correct judgment of the detection algorithm. In Divvala et al. [22], the context-based target detection is summarized, experimented and analyzed; they concluded that context information not only improves the detection performance, but also makes the residual error made by the detector more reasonable. In Zhang and Mu [23], context information was combined with target detection. An Inside-Outside Network (ION) is proposed to incorporate context information with regions of interest in Bell et al. [24]. They used context information with spatial recurrent neural networks. In the network, multiple levels and scales information are extracted by skip pooling. In Zagoruyko et al. [25], the multi-scale spatial context is attached to the region-based CNN model to extract the relationship between the target and the background. Wang et al. [26] used the multi-scale context attached to the detection feature and improved the detection performance. These methods achieved good results in some aspects of object detection, such as detection accuracy and speed. Although they provided an important reference for later researchers, they still left significant room for improvement in some respects.

### 2.3. Databases

Detection database is an important basis for object detection and an open database is an important platform for algorithm performance comparison. In the field of traffic sign detection, Felsberg and Larsson [27] and Stallkamp et al. [28] introduced detection databases. In these databases, besides providing traffic scene images, traffic sign annotating information was also included. The famous traffic sign detection database includes the German traffic sign detection benchmark (GTSDB) and the Belgian traffic sign classification (BTSC) databases. In particular, the GTSDB attracted an increasing number of researchers to find new algorithms to verify and compete, leading to some good results. Researchers of China have paid increasing attention to the problem of traffic sign detection [29,30,31,32,33], which has necessitated the construction of a traffic sign database in China. In 2016, Yang [34] constructed the Chinese traffic sign dataset (CTSD), which attracted increasing attention from Chinese researchers. As an important basis of object detection, the size of the database has a significant impact on the detection performance, however, building a large database is time and labor consuming. Therefore, using the existing databases to expand the dataset size has become very important. 

## 3. Overview of Our Method

**Figure 2:**
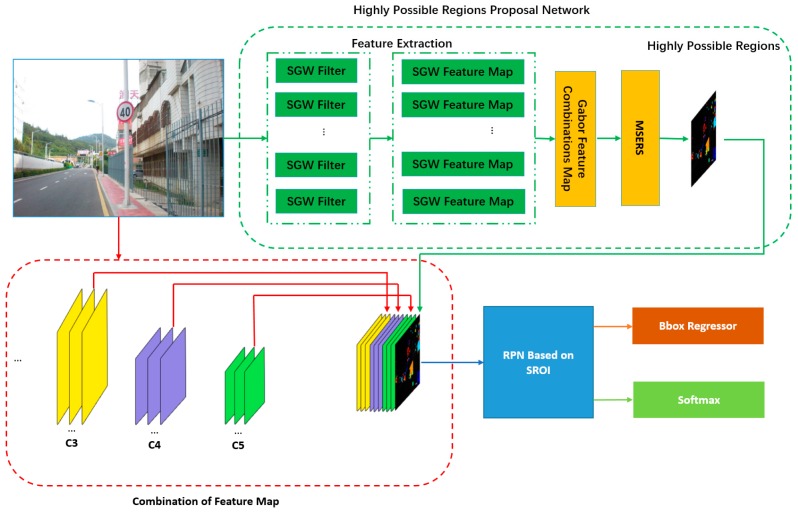
The processing flow of our method.

Our traffic sign detection and super class classification process are illustrated in Figure 2. The first part of our method is shown in the green dotted box. This part is defined as the highly possible regions proposal network. The function of this part was to propose highly possible areas of traffic signs and eliminate most non-traffic sign areas. In detail, the traffic scene image was filtered by eight simplified Gabor wavelets (SGWs), which enhanced the edge of the image and smoothed the non-edge areas. The output of the eight SGW-filtered traffic scene map was synthesized to one feature map and processed by the maximally stable extremal regions (MSERs) algorithm to get the highly possible regions. The second part of our method is shown in the remainder of Figure 2 and is similar to the Faster R-CNN algorithm [9], to which some improvements have been made. The pre-trained VGG16 is used as the feature extraction algorithm. Unlike the Faster R-CNN, which uses the fifth layer features, a combination of features from the third, fourth, and fifth layers of the VGG16 is used, inspired by Bai and Ghanem [35]. These combined features are useful for small object detection as shown in the red dotted box in Figure 2. In the regions proposal stage, regions of interest based on the information of the HP-RPN is proposed, which significantly reduced the number of candidate areas. At the stage of classifying traffic signs to super classes, secondary regions of interest information are fused, which would be helpful for the correct judgment of the algorithm.

## 4. Improved Faster R-CNN

Faster R-CNN anchor-based detection avoids the time consumption caused by sliding window detection and the detection efficiency is greatly improved without reducing the detection accuracy at the same time. Although the Faster R-CNN has achieved state-of-the-art results in common target detection areas, it has a poor recognition ability for small targets; the realization mechanism of the Faster R-CNN make it unable to recognize small targets well. Faster R-CNN faces three problems in small target detection. Firstly, after down-sampling and pooling in the convolutional layers which has a stride that is larger than 1, the small target information left in the feature layer is too coarse. For example, the VGG16 model uses the ROI-polling region from the fifth layer of convolutions (“conv5”), which has an overall stride of 16. If the traffic sign in a traffic scene image is less than 16 × 16, in the fifth convolution layer, the ROI-pooling region is less than one pixel. The advantage of original Faster R-CNN with rich feature information is no longer rich for small target detection. Secondly, in target detection, in order to avoid the introduction of redundant information and the loss of valuable information, the scale of the anchor and target often need to be matched. Faster R-CNN anchor scale for general target detection cannot be directly applied to small target detection. Finally, as the sliding window detection algorithm treats every pixel equally, in Faster R-CNN, every anchor is equal, which results in the redundancy of processing. However, it is necessary to introduce some prior information, to give some confidence to each anchor or to filter out some anchors. 

In Section 4.1, the Highly Possible Regions Proposal Network is proposed to filter part of the anchors of the original Faster R-CNN which aim to improve the speed of the process. In Section 4.2, in order to improve the accuracy, on the basis of the original Faster R-CNN which uses the fifth layer feature information of VGG16, the shallower layers feature information of VGG16 is fused. For further enriching the feature of the detection region, SROIs information is added on the ROI. 

### 4.1. Highly Possible Regions Proposal Network

As one of the categories of object detection, traffic sign detection has attracted the increasing attention of researchers. Compared with general objects detection, traffic signs are designed with strict colors and shapes so that they can be distinguished more easily from their background by human beings or computer systems. Therefore, traffic signs detection methods generally use color information, shape information, or both. However, due to natural factors such as illumination, weather and the reasons for the image acquisition sensor itself, the color and shape information in traffic sign images cannot always be stable. Therefore, it is necessary to weaken the noise and enhance the useful image features such as the color or shape by image pre-processing. For example, Sheikh et al. [36] used color restoration technology and Bahlmann et al. [37] used shape intensify technology. From a microscopic point of view, the shape of an object in an image is composed of its edges. While the low-level semantics are strengthened, the high-level semantics are usually strengthened. Compared with face detection, medical image detection and satellite image detection, the edge features of traffic signs are more obvious. In order to effectively propose regions of traffic signs, SGW is used to enhance the edges of the traffic scene image and smoothen the areas without edges in the images. In this way, the features of traffic signs can be strengthened and a foundation for post-processing can be laid.

#### 4.1.1. Simplified Gabor Filter Model

The Gabor function can extract relevant features in different scales and directions in the frequency domain. In addition, the Gabor function is similar to the biological function of human eyes, so it is often used in texture recognition and has achieved good results [38,39]. The texture representation ability of Gabor filters makes it widely used in the fields of image segmentation [40], facial expression recognition [41] and object detection [42].

Pellegrino et al. [43] found that the imaginary part of Gabor wavelet (GW) has a good edge feature extraction ability under special parameters. The imaginary part of this function is

(1) $$G\left(x,y\right)=\exp\left[-\frac{\left(x^{2}+y^{2}\right)}{2\sigma^{2}}\right]\cdot \sin\left[\omega \left(x\cos\theta +y\sin\theta \right)\right],$$

where ω is the spatial frequency and σ is the standard deviation of the Gaussian function in both directions of the X-axis and Y-axis. If the spatial frequency and standard deviation satisfy σ⋅ω≤1, the edge extraction ability of this function will be enhanced.

Because Gabor filters can only enhance the information of edges matching the scale and direction of Gabor kernels, multi-scale and multi-direction Gabor kernels are often used to weaken the loss of information and take into account the edges of different scales and directions. The method typically used is to divide the two-dimensional plane in order to set the value of θ, i.e., θ=2kπ/8, k=0,…,7. The size of the scale depends on the size of the edges in the specific application requirements. 

The traditional Gabor wavelet (TGW) with specific parameters has been widely used to extract image edge information in the field of target detection [44,45]. However, the orientation and scale of the object’s edge are often uncertain and it is impossible to extract all edge information of the targets with a certain orientation and scale of TGW. In order to take into account the edges of different directions and scales, Gabor filters with corresponding directions and scales are needed, and the increase in the number of filters means the increase of the processing time, which is unbearable in real-time applications. Therefore, although TGW has a better edge information extraction ability, traffic sign detection applications usually need real-time processing, hence, it is meaningless if it cannot solve the problem of time consumption.

In order to maintain the edge information extraction ability of TGW and reduce the processing time of the computer, simplified Gabor filters were used proposed in Choi et al. [46] to enhance the edge information of the traffic scene images. The SGW can be regarded as approximate values of traditional Gabor wavelets. SGW is a discrete value obtained by quantifying the continuous value of TGW. As described in Choi et al. [46], the SGW feature is extracted by the computer at each pixel and compared with TGW, there is no Fourier transform process, which saves a lot of time.

The results of the quantified continuous values from the TGW is defined to be quantization levels. The quantization levels selected in our method are the same as Shao et al. [47]. Because the imaginary part of the TGW is anti-symmetry, one quantization level is set to be zero, the positive quantization levels and the negative quantization levels are set to be the same. Hence, we suppose the number of positive and negative quantization levels are equal to be n. Considering the zero quantization level, there are 2n+1 values in our approach. Suppose the largest magnitude of the TGW is *M*, in order to discrete the amplitude of TGW, let i=1,…,n, so the positive quantization levels $q_{p}(i)$ and negative quantization levels $q_{n}(i)$ are defined as

(2) $$q_{p}(i)=\frac{M}{2n+1}\cdot 2i$$

(3) $$q_{n}(i)=-\frac{M}{2n+1}\cdot 2i.$$

In order to balance the processing time and efficiency, two scales and four directions of TGW are used. The $\phi_{\theta_{i},\omega_{j}}^{\prime }\left(x,y\right)$ denotes the output of the traffic scene image filtered by an SGW. The eight feature maps are synthesized into one map, denoted by $\phi_{\theta ,\omega }^{\prime \prime }\left(x,y\right)$, by finding the maximum value of the eight feature maps at each pixel using

(4) $$\phi_{\theta ,\omega }^{\prime \prime }\left(x,y\right)=\max\left\{\phi_{\theta_{i},\omega_{j}}^{\prime }\left(x,y\right),\;i=0,1,2,3\;\mathrm{and}\;j=0,1\right\},$$

The orientation selection adopts an equal half plane angle selection method so that $\theta_{j}$ belongs to (0,π) instead of (0,2π) since the SGWs with the opposite direction have the same edge extraction capacity, thereby reducing the filtering time to half of the original. As the direction of the simplified Gabor kernel divides the two-dimensional plane during the same step, it balances every direction. Edges are the expression of local information of the image and the purpose of feature transformation to extract the local information, thus, the size of the SGW should not be too large. In our method, the best size of the window, found by experiments, is 5×5 pixels.

**Figure 3:**
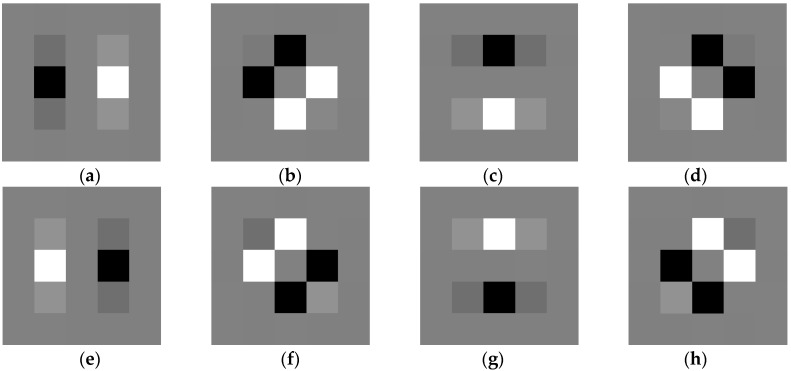
The eight simplified Gabor wavelets (SGW) filters with different parameters. (**a**) $\theta =0,\;\omega =0.3\pi ;\;\left(b\right)\;\theta =\frac{\pi j}{4},\;\omega =0.3\pi ;\;\left(c\right)\;\theta =\frac{\pi j}{2},\;\omega =0.3\pi ;\;\left(d\right)\;\theta =3\pi j/4,\omega =0.3\pi ;$ (**e**) $\theta =0,\omega =0.5\pi ;\;\left(f\right)\;\theta =\frac{\pi j}{4},\omega =0.5\pi \;;\;\left(g\right)\;\theta =\frac{\pi j}{2},\omega =0.5\pi ;$ and (**h**) $\theta =\frac{3\pi j}{4},\omega =0.5\pi$.

**Figure 4:**
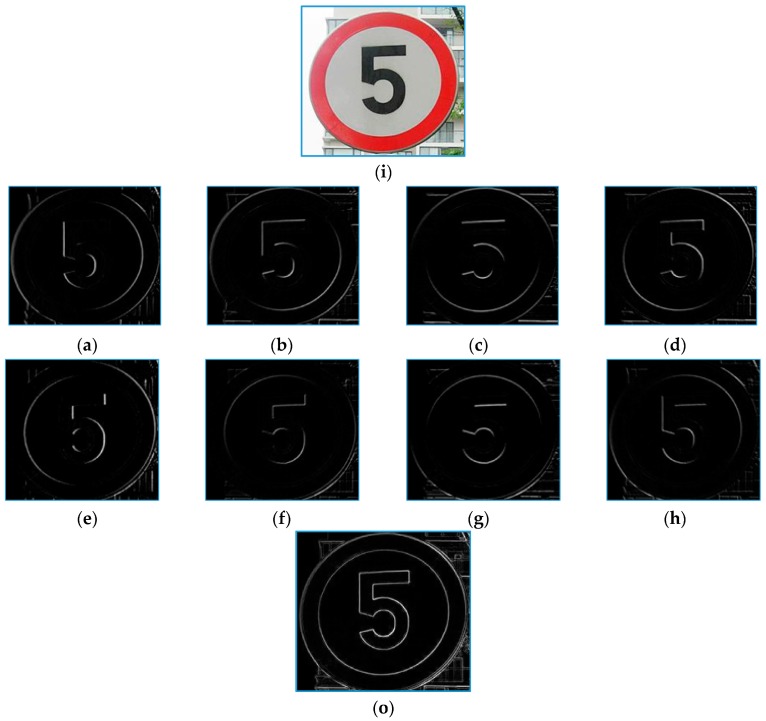
The sample processing by the SGW wavelets and the synthetize feature map: (**i**) the input image; (**a**–**h**) filtered by kernels corresponding to Figure 3, and (**o**) synthetize feature map.

The eight SGW filter kernels are shown in Figure 3. The first and the second rows of Figure 4 show the example of the result of a speed limit sign convoluted with the eight SGW filter kernels corresponding to Figure 3. The third row shows the result of the synthesis of the eight filtering maps into one map by Formula (4). From frames a–h of Figure 4, the direction of the edge of the traffic sign that coincided with the SGW filter was enhanced. Furthermore, all the edges of the traffic sign synthesized by the filtering maps were strengthened in the last image. From the above, a conclusion can be drawn that the SGW could, in fact, enhance the edges of images while reducing the processing time. 

#### 4.1.2. Maximally Stable Extremal Regions

The maximally stable extremal regions (MSERs) algorithm is a commonly used image blob detection method and was first proposed to solve such problems in Aghdam et al. [48]. This method is widely used in text detection, trademark detection and traffic sign detection. The MSERs algorithm has been used to extract the region proposal from the color probability map [49] and to detect the traffic signs on a gray-scale map [50]. These methods have good results in the regional proposal, but based on different feature maps, the proposal results of the MSERs algorithm are often quite different. Therefore, it is necessary to optimize the image features before MSERs processing. 

Inspired by the watershed algorithm, MSERs obtain a set of binary images segmented by a series of thresholds. Then the connected regions between adjacent threshold images are analyzed and MSERs are finally obtained. When the SGW feature map is separated by a threshold, the value of the pixel larger than the threshold is set to 1 and the pixel smaller than the threshold is set to 0. MSER is the areas where the shape stability is maintained over a wide threshold range. Our previous research [47] showed that SGW-processed synthetic feature maps could help to enhance the edge information of traffic signs and smooth the interior non-edge areas of traffic signs. According to the mechanism of MSERs, this will facilitate the appearance of traffic signs in the proposal regions. Therefore, in this paper, MSERs based on the SGW feature map is also applied for the region proposal in order to form a high possible region proposal network.

**Figure 5:**
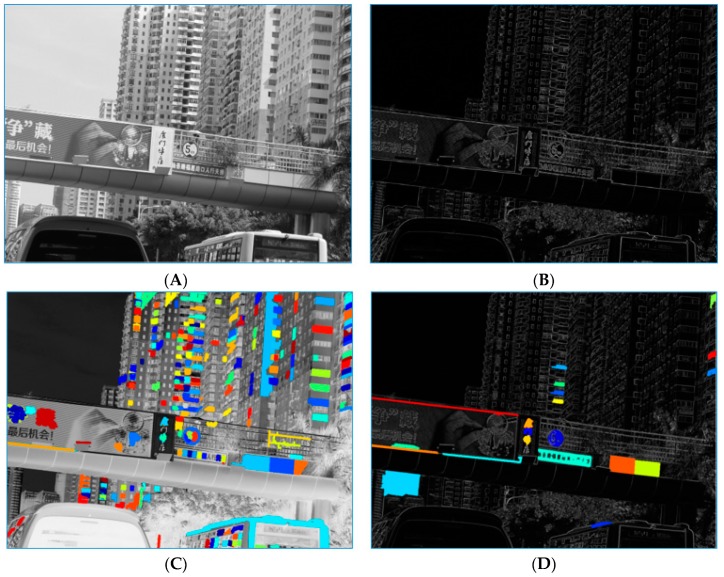
The process of finding the highly possible regions. (**A**): grayscale image, (**B**): feature map by synthesis the eight SGW feature maps, (**C**): maximally stable extremal regions (MSERs) on the frame (A), (**D**): MSERs on the frame (C).

In Figure 5, examples of a traffic scene image processed by HP-RPN is shown. Image A in this figure is the gray-scale image of the traffic scene, image B is the output of a synthetic map using the eight-SGW filtered map, image C is the result processed by MSERs on the gray-scale image, and image D is the result of processing by MSERs on image B. As shown in this figure, the highlighted regions are less numerous than those in image C and the areas where the traffic signs are located remain. This is a preliminary demonstration of the HP-RPN’s capabilities. Compared with our previous research [47], which used the SGW features in the areas of MSERs to classify traffic signs, in the current paper, the SGW + MSERs are simply used for regions proposal and the features useful for classification are the pre-trained features of the VGG16.

### 4.2. Detection Features Enrich

#### 4.2.1. Shallower Layers Feature Fusion 

In order to use the excellent detection performance of the Faster R-CNN and give it the ability to detect small targets, some improvements are made to the Faster R-CNN. Rather than relying on the fifth layer, the features from some of the shallower layers are used. In order to make full use of the information of the VGG16 feature layers, inspired by Ren [9], the features combined from layers res3d, res4f, and res5c of ResNet-50, the features of the third, fourth, and fifth layers of VGG16 are used as the input to the RPN of the original Faster R-CNN. Similar to Bai et al. [35], the third layer is down-sampled and the fifth layer is up-sampled with a stride of 2. For an input image size of 224 × 224, the size of the feature map of the fourth layer would be 14 × 14. After down-sampling the third layer and up-sampling the fifth layer, respectively, the output feature maps of the two layers were all of size 14 × 14. These three feature maps are synthesized to make them into a deeper matrix of 14 × 14. The synthesized feature map is used as the input of the RPN, which is shown in the red dotted box of Figure 2. Such processing not only retained better feature expression of the large traffic signs on the fifth layer, but also extracted a greater quantity of small traffic sign feature information from the third and fourth levels and provided the information basis needed for later detection and recognition.

#### 4.2.2. Secondary Region of Interest

The human brain has an excellent detection and recognition ability and can detect and recognize targets quickly even when the targets and backgrounds are complex. It can effectively detect targets under difficult situations such as unideal lighting, deformation of objects and partial occlusion. Importantly, the human brain also has the powerful ability to learn and inference. It can detect and recognize targets not only based on the information of the target but also based on the information of the environmental context. Inspired by the ability of the human cognitive system to detect and recognize objects, many scholars have attempted to imitate the human visual system to improve the ability of computer image detection and recognition.

Similar to humans, computer object detection aims to detect the size and position of objects from the background which contains the objects. Most traditional detection feature extraction methods have been based on the target itself and the detected information have been fully based on the target region. There is a strong connection between the target and its environment, which is called context information. Compared with recognizing the target through the information of the target itself, other objects around the target can be paid attention to because the specific environment will provide a rich context for the detection system. In the process of target detection and recognition, the full use of context information has been adopted by some new methods. These have introduced scene information and the mutual constraints of targets into target detection and classification, which can improve the performance of detection and classification, and help reduce uncertainty.

**Figure 6:**
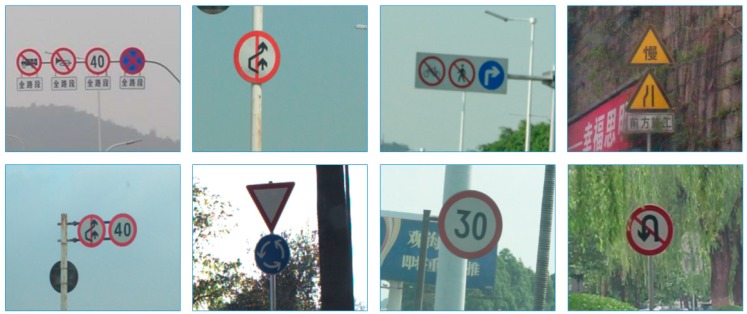
Examples of traffic signs with contextual information including other traffic signs, poles, etc.

In practice, a scene that includes targets is often very structured, especially for manually designed objects such as traffic sign, whose structure is often more obvious. Structural information often has a great impact on the detection result. In Figure 6, the components that fix traffic signs to structures and objects near the traffic signs often help us to judge whether or not they are traffic signs. Such examples include metal or cement pillars for fixing traffic signs, other traffic signs beside the target one, buildings next to traffic signs, and the road environment of traffic signs. All of this information can help one determine that the detected object is indeed a traffic sign.

**Figure 7:**
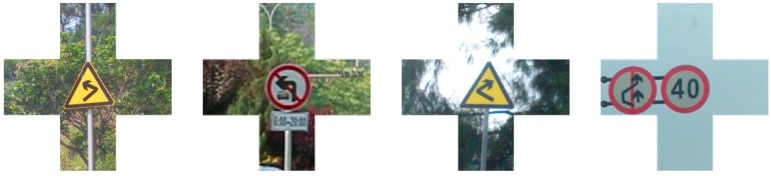
Traffic signs with cross-shaped secondary regions of interest.

As shown in Figure 7, in addition to the information on the traffic signs, a cross-shaped pattern also contains the structural information of the traffic signs. From human visual habits, this structural information is conducive to the detection of traffic signs. In most cases, the structural information is usually above, below, to the left and to the right of the traffic signs. In order to reduce the influence of redundant image information on the complexity of network structure and maximize the proportion of valuable information in the training samples, the four corners of image information were removed. Intuitively, such a method can be looked on as directly using the cross-shaped images to train the detection network. Compared with the traditional ROI, the regions outside the center area of the cross-shaped image are defined as the secondary region of interest (SROI). At the same time, the markers of the training sample database and the test sample database are expanded and the markers of the four SROIs to every traffic sign in the database are added. 

**Figure 8:**
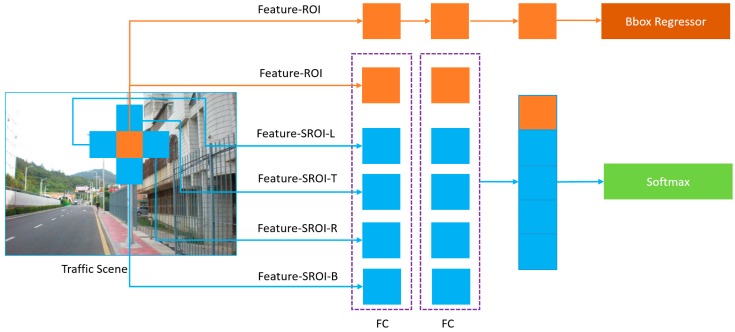
The feature combination of the region of interest (ROI) and secondary ROIs (SROIs). The L, T, R, B refers to the $SR_{L}$, $SR_{T}$, $SR_{R}$, $SR_{B}$, respectively.

In order to effectively utilize the information of the SROIs defined, an SROI extraction algorithm is added to the original Faster R-CNN. The structure is shown in Figure 8. The ROI proposal is the same as with the original Faster R-CNN when a traffic scene image I(x,y) was input into the network, the Faster R-CNN extracts a number of proposal regions as ROIs by the combination features of the third, fourth, and fifth layers of the VGG16. The ROI is expressed as $R=(x_{l},y_{t},x_{r},y_{b})$, where $\left(x_{l},y_{t}\right)$ represents the pixel in the top left corner and $\left(x_{r},y_{b}\right)$ represents the bottom right corner of the ROI.

After the ROI was extracted, the novel SROIs is added based on the previous feature map. In the SROI extraction process, four SROIs are added to each ROI. The four SROIs were defined as the left, top, right, and bottom SROIs, denoted as $SR_{L}$, $SR_{T}$, $SR_{R}$, $SR_{B}$, respectively. The four SROIs are defined as

(5) $$SR_{L}=(x_{l}-w,y_{t},x_{l},y_{t}-h)$$

(6) $$SR_{T}=(x_{l},y_{t}-h,x_{r},y_{t})$$

(7) $$SR_{R}=(x_{r},y_{t},x_{r}+w,y_{b})$$

(8) $$SR_{B}=(x_{l},y_{b},x_{r},y_{b}+h),$$

where w and h represent the width and height corresponding to the ROI. With these four equations, the features of each SROI can be obtained.

Figure 8 shows the process of how the SROIs information is combined with the ROI for detection. In this figure, the area covered by the orange rectangle was the ROI that was consistent with the Faster R-CNN. The four areas of $SR_{L}$, $SR_{T}$, $SR_{R}$, $SR_{B}$ were the defined SROIs. What should be emphasized is that in our approach, the SROI is used only as decision information for classification by the softmax function together with the ROI. The bounding box regressor was based on the information of the ROI, which was the same method used in the Faster R-CNN. If these five boxes were regressed independently, they would overlap or disperse; however, the area of concern was the ROI that corresponded to the traffic signs marked by the database, and the SROIs only provide auxiliary decision information for super class classification. 

### 4.3. Loss Function

In our method, the Loss function is similar to the original Faster R-CNN [9], but because our method has an anchor screening process, it is different from the original Faster R-CNN. The loss function is defined in Formula (9), the loss function realizes two functions, one is to adjust the category of the detected object and the other is to adjust the position of the bounding box.

(9) $$L(\{p_{i_{HPR}}\},\{t_{i_{HPR}}\})=\frac{1}{N_{cls}}\sum_{i}L_{cls}(p_{i_{HPR}},p_{i_{HPR}}^{*})+\lambda \frac{1}{N_{reg_{HPR}}}\sum_{i}p_{i_{HPR}}^{*}L_{reg}(t_{i_{HPR}},t_{i_{HPR}}^{*})$$

here, $i_{HPR}$ is the index of an anchor in the highly possible regions of a mini-batch and $p_{i_{HPR}}$ is the predicted probability of the anchor $i_{HPR}$ being an object. The ground-truth label $p_{i_{HPR}}^{*}$ is 1 if the anchor is positive and is 0 if the anchor is negative. $t_{i_{HPR}}$ is a vector representing the 4 parameterized coordinates of the predicted bounding box in the highly possible region and $t_{i_{HPR}}^{*}$ is that of the ground-truth box associated with a positive anchor. $L_{cls}$ is the classification loss over two classes (object and not object). $L_{reg}(t_{i_{HPR}},t_{i_{HPR}}^{*})=R\left(t_{i}-t_{i}^{*}\right)$ is the regression loss and R is the robust loss function. In our case, $N_{cls}$ is the size of a mini-batch and $N_{reg_{HPR}}$ is the number of anchor locations in the highly possible region. λ is a balancing parameter.

The improvement of the loss function of the original Faster R-CNN is mainly reflected in the different number of anchors that the loss function needs to traverse. The influence of the prior information of HP-RPN on the network is realized by the loss function. In the original Faster R-CNN, the number of anchors is 14×14, which corresponding to the $N_{reg_{HPR}}$ of our loss function, the number is fixed. The number of anchors in the $N_{reg_{HPR}}$ of our method is the number of anchors covered by MSERs and the anchors contained the MSERs. Through this mechanism, the number of anchors that the loss function needs to traverse will be greatly reduced. Then it improves the speed of object detection. Importantly, $N_{reg_{HPR}}$ can be used as an a priori information entry, providing possibilities for future a priori information based detection algorithm optimization.

## 5. Experiments and Analysis

In this section, a brief introduction is first given to the databases of German and Chinese traffic scenes and the method that is used to merge the two databases. Then, the metrics method used in our paper is defined. Based on these metric methods, the process speed improvement by our HP-RPN and the detection accuracy optimization after the detection features are enriched in our method are evaluated. Finally, some examples of the results of our detection algorithm are shown.

### 5.1. Experimental Dataset and Computer Environment 

**Figure 9:**
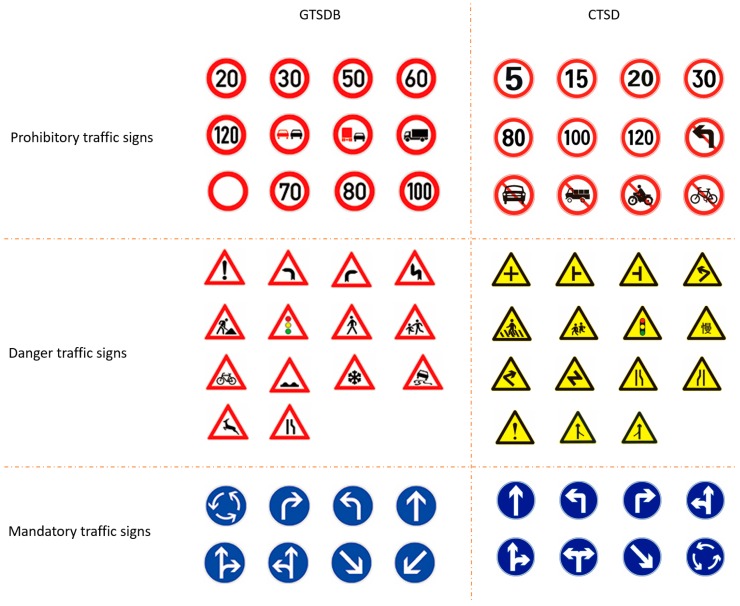
Examples of subclasses in the German traffic sign detection benchmark (GTSDB) dataset and the Chinese traffic sign dataset (CTSD). Merging the Prohibitory, Danger and Mandatory traffic signs from the two databases into super classes, respectively.

In our detection methods, the publicly available Chinese traffic sign dataset (CTSD) dataset and the German traffic sign detection benchmark (GTSDB) dataset were adopted for the performance evaluation. In both the CTSD and GTSDB, there are three classes of traffic signs, namely, danger, mandatory, and prohibitory. In this paper, those three classes are defined as super classes and each traffic sign is defined as a sub-class. All the data in the two databases are in the form of images which were captured in real traffic scenes by on-vehicle cameras. Figure 9 shows examples of the subclasses of the CTSD and GTSDB.

The GTSDB dataset includes 900 high-resolution natural traffic scene images in Germany. There are 1213 traffic signs in the whole database and in each image, there are 0 to 6 traffic signs. The size of every traffic sign in this database varies from 16 × 16 pixels to 128 × 128 pixels. For each image, the size is 1360 × 800 pixels. These images are divided into two parts, the first part is the 300 testing images, and the second part is the 600 training images. In the test dataset, there are 63 danger signs, 49 mandatory signs and 161 prohibitory traffic signs, respectively. 

The detection performance of traffic signs in China is validated on the Chinese traffic sign dataset (CTSD) in Yang et al. [34]. The sizes of the images in this database are either 1280 × 720 or 1024 × 768. There are in total 1100 images in the CTSD, 700 images for training and 400 images for testing. The traffic signs include danger traffic signs, mandatory traffic signs and prohibitory traffic signs. The test database included 129 danger signs, 139 mandatory signs and 264 prohibitory traffic signs, respectively.

Compared with the demands of training the Faster R-CNN, the number of samples is insufficient for both the GTSDB and CTSD. Too few training samples would easily lead to the overfitting of the network. To solve the problem of sample shortage, the two databases are merged into one because the detection results of our detection network are the super classes of traffic signs. In other words, the traffic signs are detected and classified as prohibitory, danger, or mandatory signs in super classes. Because the shape of the same super classes of the two traffic sign databases is the same, the same super class of the two databases is merged into one class to form a larger traffic sign detection database. The database merging method is shown in Figure 9.

Since our training data includes the information of the SROI region and there is no SROI mark information in the two databases, four SROI region markers are added to each image of the two databases based on the original data markers. These four regions are rectangles of the same size as the original data markers areas and they extend in four directions (left, right, above, and below) away from the original region, and the adjacent edges are coincidents. The four regions belong to the same category as the central rectangular region. The calculation methods of the four SROIs is defined in Equations (5)–(8).

Our traffic sign detection methods were experimented in the MATLAB software. The running platform was Windows 10 64-bit operating system, Intel^®^ HD Graphics 520 Graphics, 8 GB DDR3 memory, Intel(R) Core^(TM)^ i5-6300U, CPU@2.40 GHz. Our algorithm runs on a CPU. In order to save processing time, the HP-RPN and VGG16 feature extraction are executed in parallel by two-thread programming.

### 5.2. Evaluation Metrics

In the field of image classification, the accuracy and recall rate are often used to measure the classification performance. However, in the field of target detection, if the detector detects an object without the information about its location in the image, it has little use. Since the position of the object in the image must be predicted and it is difficult to predict the position consistent with the position of the ground truth, measurement methods must be used to accurately measure the prediction results. Our evaluation indicators, therefore, include the intersection over union (IoU), average precision (AP), mean average precision (mAP), and precision indexes. 

For current detection algorithms, it is impossible to make the detected area exactly the same as the ground truth, partly because of the accuracy of the detection algorithm, and partly because the subjectivity of the database markers means that the ground truth may not be exactly the “truth”. In the field of target detection, the IoU is often used to measure the accuracy of detection, which refers to the degree of coincidence between the detected area and the ground truth. The IoU is defined as follows:

(10) $$I\mathrm{oU}=\frac{area(B_{\det}\cap B_{gt})}{area(B_{\det}\cup B_{gt})}\times 100\%$$

In the equation above, $B_{\det}$ refers to the detection bounding box, $B_{gt}$ refers to the ground true bounding box, $area(B_{\det}\cup B_{gt})$ indicates the union area of the detection area and the ground truth. In our paper, if the IoU is larger than 50%, this will be identified as a correct detection. 

In order to comprehensively measure the accuracy of the test, the precision and recall rates are used. The precision and recall rates are based on three indicators: the false positive (*FP*), the true positive (*TP*) and the false negative (*FN*). *TP* and *FP* refer to the ratio of correctly and falsely detected objects in all region proposals. FN refers to the number of regions which include objects that should be detected but are not proposed. The definitions of precision and recall rate are

(11) $$Precision=\frac{TP}{FP+TP}$$

(12) $$Recall=\frac{TP}{FN+TP}.$$

### 5.3. Performance of Highly Possible Regions Proposal 

**Figure 10:**
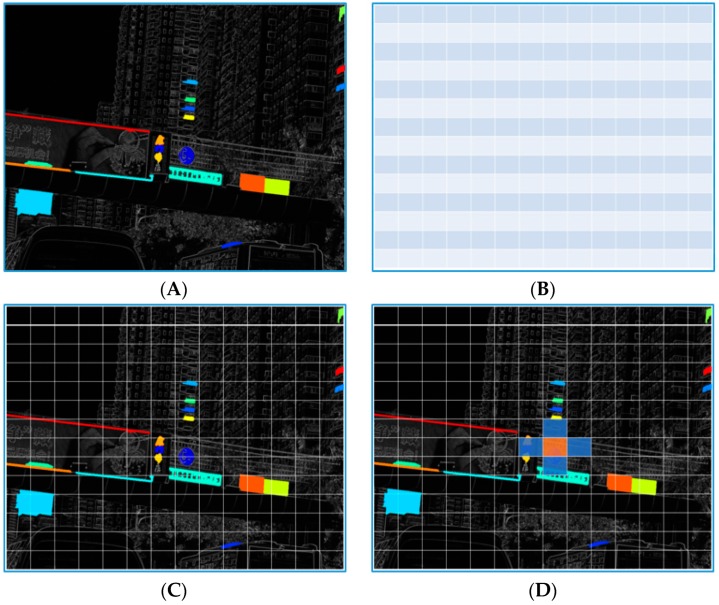
The process of filtering the ROIs of a faster region with a convolutional neural network (R-CNN) with MSERs. (**A**): regions proposal by MSERs, (**B**): anchor points of Faster R-CNN, (**C**) MSERs and Faster R-CNN coincidence proposal regions, (**D**): ROI with SROI information.

In Figure 10, the process of filtering the ROIs of Faster R-CNN with MSERs is shown. Image A in this figure is the map processed by MSERs on the synthetized eight SGW filtered map. Image B is the feature matrix of the combined features of the third, fourth, and fifth layers of VGG16. To reduce the number of detection windows in the original Faster R-CNN, most of the almost impossible areas are filtered out. The filtering method is used to find the ROIs which include the MSERs from the HP-RPN of our method. In this way, it is able to filter out more than half of the ROIs compared to the original Faster R-CNN. The number of regions of interest remaining will be less than the number of ROIs from the Faster R-CNN because more than half of the ROIs were not included in the MSERs. Compared with Reference [9], based on the prior information of MSERs, the credibility of the proposed regions can be improved, the number of proposal regions can be greatly reduced, and the processing time can be further saved.

As shown in image D of Figure 10, the light-yellow area mainly covers the traffic signs. However, because they were not fully covered, the bounding box needed to be constantly adjusted. This problem originates with the anchor-based regression method, which aims to avoid the high time requirement of the sliding window target detection method, but in which the choice of anchor points is fixed without any prior knowledge. In order to correct the coverage area, the bounding box must be constantly regressed and rectified, which is a highly time-consuming process. Clearly, with the information of SROIs (shown as the light blue area in image D), the traffic sign information is fully expressed. Therefore, our SROI-based feature representation would have obvious advantages for such samples. The number of samples is not small because the location of traffic signs falling into the anchor point is random and the probability of the area corresponding to the anchor point is low.

In order to demonstrate the regional proposal capabilities of our HP-RPN, that is, showing the SGW filtering is conducive to the discovery of effective MSERs and that our method suppresses the number of negative samples, our method is compared with MSERs on a grayscale map. As shown in Table 1, the average numbers of proposals for the grayscale inputs from the GTSDB and CTSD are 388 and 321, respectively, equivalent to recall rates of 97.1% and 98.12%. The average numbers of proposals of the SGW Map + MSERs on the two databases are 276 and 178, respectively, with recall rates of 99.63% and 99.62%. The number of proposal regions from our method is less than from the grayscale + MSERs approach, and the recall of our method is higher, which shows that our method can improve the recall rate while restraining the number of region proposals.

Compared with grayscale + MSERs, which took 40 and 38 milliseconds per image on the GTSDB and CTSD, respectively, our method takes several more milliseconds because our method includes the SGW filtering process rather than using the grayscale image directly. However, compared with the main part of the Faster R-CNN, the time consumption of the HP-RPN network is lower and these two parts are logically executed in parallel. With parallel programming, the increase in time for the SGW map will not affect the final system processing time.

The average number of proposals, recall rate, FNs, and time cost on GTSDB + CTSD was calculated from the experimental results of GTSDB and CTSD. Since our HP-RPN does not have the ability to learn and remember, its performance will not change with an increase in the sample size. The performance advantages of our method can also be seen from the results.

**Figure 11:**
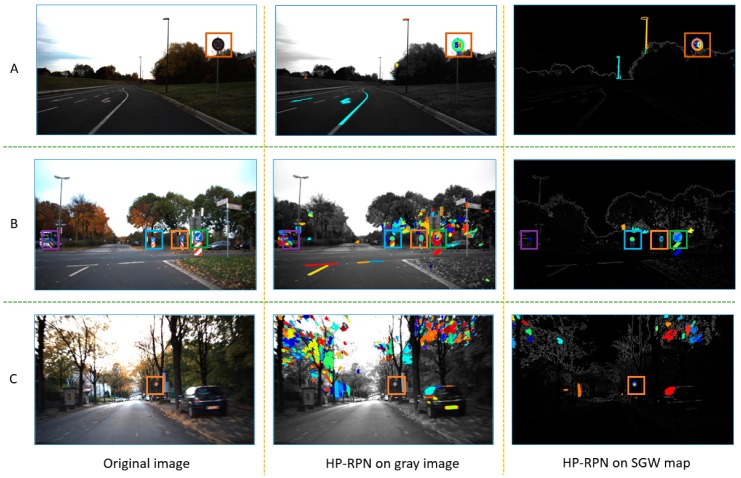
Examples of regions proposal by the highly possible regions proposal network (HP-RPN) on a grayscale image and the HP-RPN on an SGW feature map under different situations of traffic scenes. (**A**): clear and simple traffic scene, (**B**): complex traffic scene, (**C**): motion blurred complex traffic scene.

Figure 11 shows the examples of the regions proposed by MSERs on the grayscale image and SGW feature map. The first column of this figure is the original traffic scene images, the middle column is the result of HP-RPN based on the grayscale image and the last column is the HP-RPN based on the SGW feature map. In these images of this figure, the region covered by the color square is the region where the traffic sign belongs. In the same line, the square of the same color represents the same traffic sign in the traffic scene.

Line A shows the regions proposal with clear traffic signs and relatively simple traffic scenes. From the results, it can be seen that both methods have good regions proposal results. What is good here is that the regions where the traffic signs belong to is proposed and the total number of proposal regions is small. In line B, although the four traffic signs are proposed in both methods, it is obvious that the number of regions proposed by HP-RPN based on SGW is significantly less than that by HP-RPN based on grayscale images. The reduction of the number of proposed regions will help to filter anchors and then improve the processing speed of the system. The last line is motion blurring, small targets and complex traffic scenes. Because the traffic sign is small and blurred, in the process of HP-RPN based on a gray image, the region of the traffic sign is not successfully proposed, but in the process of HP-RPN based on SGW, this difficulty is overcome. The experimental results show that the SGW filter has the ability to stabilize the unstable region and better the regions proposal ability.

### 5.4. Performance of Detection Features Enrich 

In addition to the number of anchors affecting the detection speed, there are two factors that have a greater impact on the detection accuracy. The first one is the matching degree between the scale of the bounding box and the detected target. The closer the two scales are, the easier it is to detect the target. The second is the feature expression of the detected region. The better the feature expression, the easier the classification algorithm can judge whether the detected region contains the target object. In this section, our method about the selection of the bounding box scales and detection features enrichment will be validated and analyzed through experiments.

#### 5.4.1. The Selection of Bounding Box Scales

**Figure 12:**
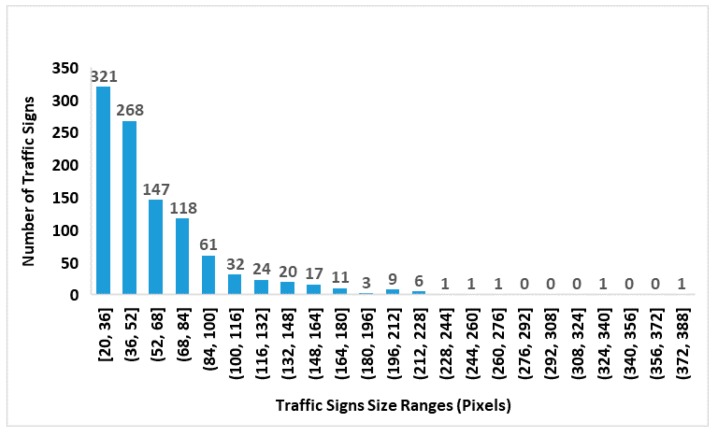
A histogram of the traffic sign size distribution in the Chinese traffic sign dataset (CTSD).

**Figure 13:**
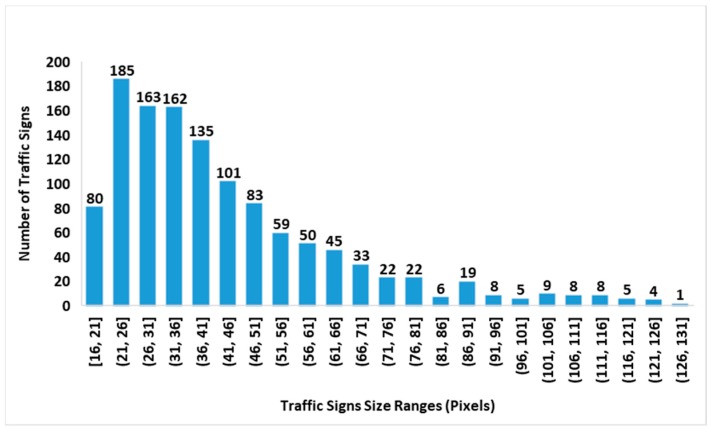
A histogram of the traffic sign size distribution in the German traffic sign detection benchmark (GTSDB).

**Figure 14:**
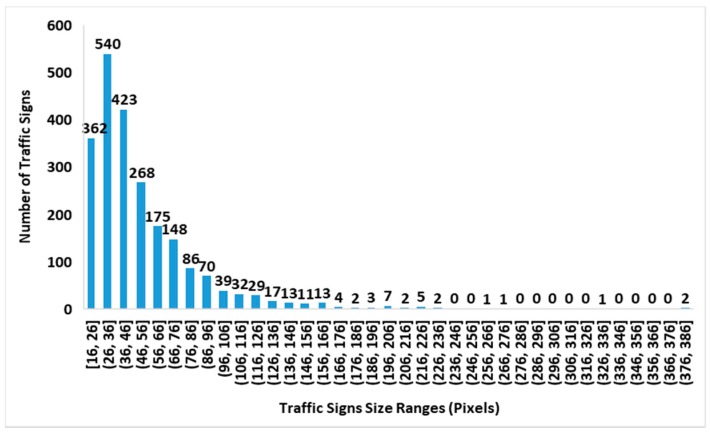
A histogram of the traffic sign size distribution in the joint GTSDB and CTSD dataset.

The distributions of traffic sign size in the GTSDB and CTSD are shown in Figure 12 and Figure 13, and the distribution of the traffic sign size in the joint GTSDB + CTSD dataset is shown in Figure 14. Since the lengths and widths of most traffic signs in the database are not equal, the maximum lengths and widths of traffic signs as the scale of the traffic signs are taken for statistics. In the joint database, GTSDB + CTSD, there are 902 traffic signs with scales smaller than 36 pixels, which are defined as small-sized; 692 traffic signs have scales between 36 and 66 pixels, which are defined as medium-sized; and 662 traffic signs have scales bigger than 66, which are defined as large-sized. Compared with the GTSDB and, especially, the CTSD, by merging the two traffic databases, the unbalanced distribution of traffic signs was alleviated to a certain extent, which will be conducive to the training of the network.

**Figure 15:**
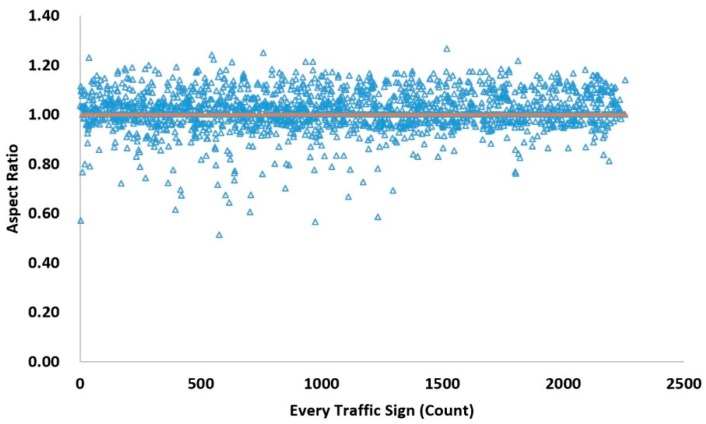
The distribution of aspect ratios of traffic signs. Each blue triangle represents a traffic sign and the orange straight line represents the average aspect ratio of all the traffic signs in the GTSDB and CTSD dataset.

In the selection of the bounding box, according to the statistics of traffic sign scale in GTSDB + CTSD, three sets of scales are used in our experiments to find suitable scales for traffic sign detection. These three sets of scales are {16^2^ 64^2^ 128^2^} pixels, {64^2^ 128^2^ 256^2^} pixels, and {128^2^ 256^2^ 512^2^} pixels. Although traffic signs have some of the common problems found in the field of image detection, such as tilt, distortion, and rotation, for the vast majority of normally installed traffic signs, the degree of deformation in images is generally not too large. As shown in Figure 15, most of the aspect ratios of the traffic signs are distributed between 0.6 to 1.3, with an average aspect ratio of 1.01, shown as the horizontal orange line in Figure 15. Therefore, in our method, the aspect ratios are set to 1:1, 1:2 and 2:1, there are, in total, 9 scales for each anchor which is the same as in Reference [9].

Table 2 shows a comparison of the detection results for different methods. mAP refers to the mean average precision when the IoU was constrained to 0.55, 0.6, 0.65, 0.7, 0.75, 0.8, 0.85, 0.9, and 0.95. The evaluated method is the same as that in Reference [9]. These detection methods can be divided into two categories. The first is the original Faster R-CNN, based on the fifth layer features of VGG16. It mainly relates to the detection performance of the original Faster R-CNN with different bounding box scales for small target detection. As the scale decreases, the detection performance of the large traffic signs decreases because, in the process of scale reduction, the bounding box cannot surround the key information of the large traffic signs. The detection performance of small-scale traffic signs has not been greatly improved by the reduction of the bounding box scale. Although the size of the bounding box changed, the traffic sign size does not change in the traffic scene. As mentioned above, the features of small traffic signs mapped in the fifth layer of the VGG16 are too coarse for detection. 

The second method of comparison is the modified Faster R-CNN that uses features combined from layers three, four, and five of VGG16 with or without the SROI information. For the methods without SROI information, as the scale of the bounding box decreased, the detection performance of the large traffic signs was slightly reduced. After analysis, this is because the largest of the three bounding boxes on the smaller scales cannot completely cover the large traffic signs, clearly reducing the detection effect. As shown in Table 2, with the decrease of the bounding box size, especially when using the {16^2^ 64^2^ 128^2^} pixels scale, the detection performance of small traffic signs was significantly improved. This shows that by fusing the third, fourth, and fifth layer features of VGG16, the detection of small traffic signs is indeed improved when using the bounding box with scales of {16^2^ 64^2^ 128^2^} pixels. 

More interestingly, when the SROI information is fused in the detection network, the detection performance of small, medium, and large traffic signs was significantly improved. Moreover, the performance of large traffic sign detection was clearly improved. Two conclusions can be drawn. First, for small traffic signs, adding the SROI context information is conducive to improving the detection performance. Second, for databases with a large range of target scale changes, when the size of the detection bounding box cannot be fully taken into account, the problem caused by the bounding box being slightly smaller than the detection object can be remedied by adding the SROI information. 

#### 5.4.2. Performance of Features Fusion

In order to further verify the effectiveness of our method in the convolution feature layers selection and SROI information fusion for target detection, the performance of the three detection methods is compared on small, medium and large traffic signs from the GTSDB and CSTD databases. The first method is the original Faster R-CNN which used the features of the fifth layer of VGG16; the second one is our method without SROIs information support and the third one is our method with SROIs. The last two methods use the features of the third, fourth and fifth feature layers of VGG16. 

**Figure 16:**
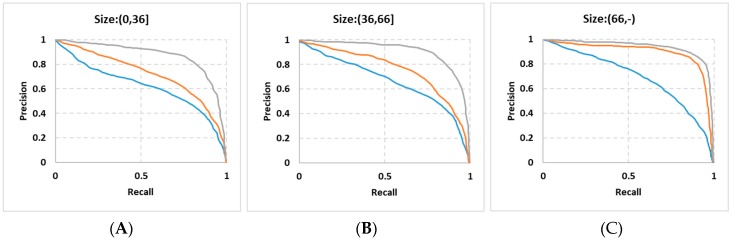
The Precision-Recall Curves, (**A**) on small traffic signs, (**B**) on medium traffic signs, (**C**) on large traffic signs; the blue lines represent the method of the original Faster R-CNN, the orange lines represent our method without SROIs information support, the gray lines represent our method with SROIs.

Figure 16 shows the PRCs (Precision Recall Curve) of the three methods on the small, medium and large traffic signs. As shown in Figure 16A–C, the blue lines which represent the original Faster R-CNN method does not perform well in the detection of these three types of traffic signs, although it is slightly better in the detection of large traffic signs. This result further confirms that the original Faster R-CNN is not suitable for small target detection. The orange lines which represent our method without SROIs have better performance because the selection of multi-layer features offsets the roughness of the deep features. Through the fusion of SROIs information, our method gets the best detection performance as shown by the gray lines. The experimental results show that the detection performance of the small, medium and large traffic signs can be improved by fusing shallower layers convolution features and adding SROI information. Experiments also show that the effective detection region feature expression plays an important role in improving the object detection performance.

### 5.5. Overall Processing Speed and Accuracy 

#### 5.5.1. Anchor Filtering

The main time consumption of object detection algorithms based on the bounding box mode is the traversal pattern of the bounding box. Unlike the sliding window based target detection methods which need different scales of windows to traverse the whole picture at each pixel, and the computational cost being unacceptable, Faster R-CNN uses an anchor-based detection method which greatly improves the detection efficiency. The dimension of this feature layer used in our method is 14 x 14. If the original Faster R-CNN structure is used, there are 196 anchors. The number of anchors is fixed and has nothing to do with the state of the image or the network. In our method, the HP-RPN regional proposal information is used to filter part of the anchors and only anchors covered by MSERs are retained, or anchors containing MSERs; all the anchors that do not intersect with MSERs are removed.

**Figure 17:**
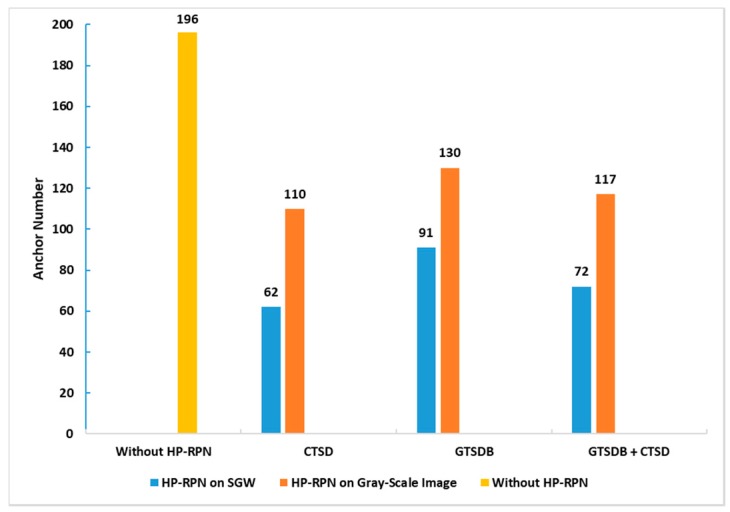
The comparison of the number of anchors of the Faster R-CNN without and with HP-RPN on different databases.

Figure 17 shows the comparison of the number of anchors of Faster R-CNN without and with HP-RPN. In the Gray-Scale image based HP-RPN, the average numbers of anchors after filtering the test datasets of GTSDB, CSTD and GTSDB+CSTD are 110, 130 and 127, respectively. The average numbers of anchors proposed by the SGW based HP-RPN are 62, 91 and 72 respectively. The numbers of the anchors of the above two results are obviously superior to the original Faster R-CNN structure, which has 196 anchors. From those results, two conclusions can be drawn: firstly, Faster R-CNN using the output of HP-RPN as prior information can indeed reduce the number of anchors; secondly, HP-RPN based on SGW is better than HP-RPN based on gray images. Importantly, the reduction in the anchor number is the key to improving the processing speed of our method. 

**Figure 18:**
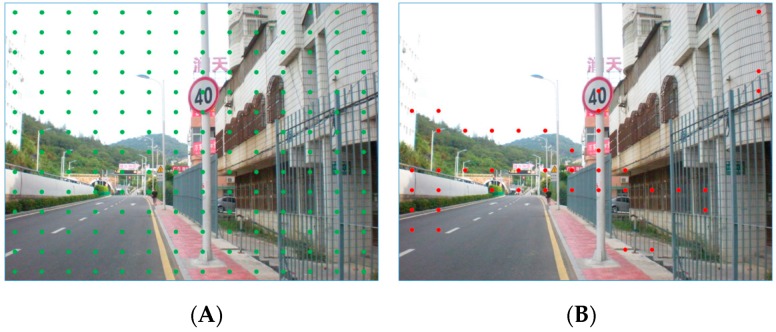
Example of anchors points of the Faster R-CNN without and with HP-RPN. (**A**) anchor points of original Faster R-CNN, (**B**) anchor points of our method.

Figure 18 shows an example of the actual anchor points without and with HP-RPN information support. Image A of this figure shows the anchor points of an input image of the original Faster R-CNN structure which has no HP-RPN support. Image B shows the anchor points of our method. The prior information is the MSERs proposal by HP-RPN as shown in Figure 10A. From Figure 18, it can be clearly seen that the number of anchors that our method needs to traverse is far less than that of the original Faster R-CNN structure. However, the traffic signs in this input image are still covered by anchors. 

For the object detection algorithms based on the sliding window, the relationship between each pixel is equal, so it is necessary to traverse every pixel. For the original Faster R-CNN which is based on anchor detection, the relationship between each anchor is equal and it is also necessary to traverse every anchor. However, in fact, the relationship between these pixels and anchors is not equal because some areas are “obviously not a traffic sign”. HP-RPN is used to express the information on “obviously not a traffic sign” and use this information as a priori information to provide decision support for the detection network. According to the mechanism of the algorithm, the large reduction of the number of anchors will greatly help to improve the processing speed of the algorithm.

#### 5.5.2. Analysis of Processing Time Consumption

The purpose of using the SGW filter in our method is to strengthen the edge feature of traffic signs because the edge feature is one of the important features of traffic signs. However, there are many edge enhancement algorithms such as Canny operator [51] which has a better edge enhancement ability for image edge information, and TGW [44] with specific parameters which also has a better edge enhancement ability. However, from the perspective of the application of traffic sign detection, real-time detection performance is one of the key requirements, so the time consumption of edge enhancement is one of the important factors to be considered. Compared with the TGW filter which needs FFT (Fast Fourier Transformation), SGW can be calculated independently for each pixel, which greatly reduces the filtering time. Table 3 shows the comparison of the computation times between the TGW, Canny and SGW. In this table, the numbers of additions and multiplications required by these three edge enhancement algorithms are analyzed in the filtering process. Those characteristics will directly determine the number of computations of the processor. From Table 3, it can be clearly seen that the SGW consumes the least computing times in the filtering process, which will be beneficial to its application in real-time image processing.

**Figure 19:**
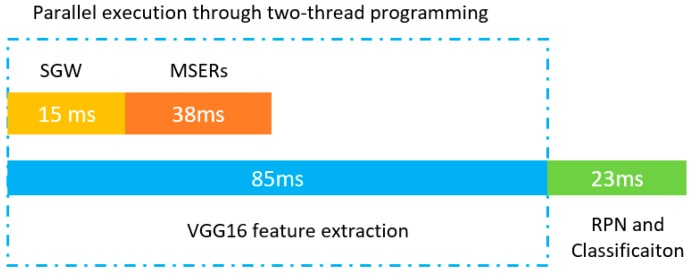
The system processing time.

When our algorithms are converted into programs, the HP-RPN and VGG16 feature transformation are executed in parallel by two thread programming. Unlike general image preprocessing, our method uses the VGG16 network to transform the image features while preprocessing the image. These two parts are processed independently through two cores of the CPU. This can offset the time required to preprocess the image. As shown in Figure 19, the average processing time of the SGW and MSERs is 15 ms and 38 ms, respectively, but the average processing time of the VGG16 feature transformation is 85 ms. Therefore, our HP-RPN network almost does not increase the whole system execution time. Our processing speed is close to 9.3 frames per second; the traffic signs can be detected when the vehicle runs for 4 m with a speed of 120 km/h. This means that if the vehicle captures the traffic sign image 200 m away from the traffic sign, when the vehicle is 196 m away from the traffic sign, the system will give the detection results. The processing speed meets the real-time processing requirements.

#### 5.5.3. Performance Comparison Analysis

Table 4 shows the effect of SROIs and multi-layer deep convolution feature information on the detection accuracy on the datasets of GTSDB and CTSD. As can be seen from this table, the larger the traffic signs, the higher the detection accuracy; this shows that the scale of the detection object directly affects the detection accuracy and the space for improving the detection accuracy mainly lies in the small targets. When the SROIs information is added to the original Faster R-CNN solitary, although small target detection has a little improvement, the performance improvement of the algorithm for large target detection is still more obvious, this may be because the feature information of the small target on the fifth layer of VGG16 is too rough. It is obvious that the detection accuracy of small targets has been greatly improved after multi-layer deep feature information fusion. From the perspective of overall detection accuracy, the fusion of multi-layer deep convolution features is better than the context information for improving the detection accuracy, this shows that the detection accuracy mainly depends on the information expression of the detection object itself. From the last two rows of this table, it can be seen that the detection achieves optimal performance by fusing the multi-layer convolution feature information and context information simultaneously. Generally speaking, the introduction of SROIs and multi-layer deep convolution feature information has greatly improved the detection performance.

Table 5 shows the comparison of the detection results of our method and other methods from References [34,52,53,54]. According to the final detection results of merging the GTSDB and CTSD databases, the detection results of our method on the three super classes are 99.53%, 98.40%, 98.44%, respectively. The total accuracy of our method on the three super classes is 99.01%. Although the detection rate of a certain super class is not the best, our detection network has a better overall detection performance than other methods. In Reference [52], the detection accuracy of both the Mandatory traffic sign and the Danger sign are 100%, but the overall accuracy is 97.44% which is lower than our method. The process speeds of our method on GTSDB, CTSD and GTSDB+CTSD are 0.111 s, 0.106 s and 0.108 s per image based on a single CPU, respectively. Compared with Reference [52] which has a processing speed of 0.130 s per image based on a GPU which is professional for image processing, our method has an obvious efficiency advantage. 

After database combination, the detection rate improves for the prohibitory and mandatory traffic signs. However, as the data show, the accuracy of the danger traffic signs has no significant improvement after the databases are combined. The reason for this phenomenon is that although the danger traffic signs in these two databases have the same triangular shape, the danger traffic signs of the GTSDB have a red border with a white interior, while those of the CTSD has a black border and yellow interior. This color difference makes it difficult for the detection network to classify the two kinds of traffic signs into the same class. 

The experimental results show that the combination of the two databases is helpful for the detection of traffic signs with the same color and shape. However, for traffic signs with obvious color differences, such as the danger traffic signs, the effect is not obvious. This shows that although a small sample size database for deep learning is useful for in-depth research, from the perspective of performance improvement, increasing the number of samples and building a larger database are obvious ways to improve the detection efficiency. The way that expanded the size of the database by merging the two databases into one is highly economical.

**Figure 20:**
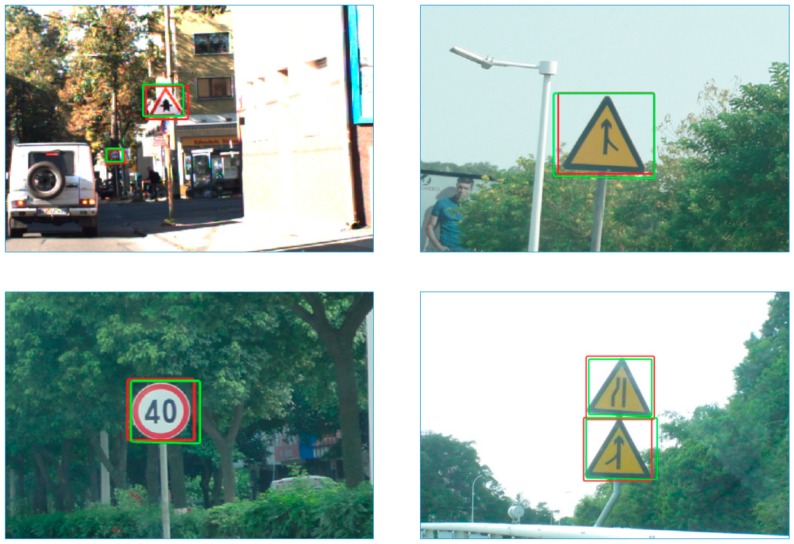
Examples of detection results. The red box represents the traffic signs mark area in the databases, the green box represents the detected areas.

Some detection result examples from the two databases are shown in Figure 20. Since traffic signs are too small in the image, just part of the picture is displayed. The red box is the traffic signs mark the area of the database and the green box represents the detected area. From the results, it can be seen that our method can accurately detect single traffic signs, multiple traffic signs, and traffic signs of different shapes. Such detection results make our methods very close to the real-time application.

## 6. Conclusions and Future Work

In this paper, to solve the problem of traffic sign detection in the natural environment, a new traffic sign detection method was proposed. A highly possible regions proposal network is proposed as a prior information provider to reduce the number of anchors produced by the original Faster R-CNN, which further reduces the processing time. Moreover, the processing time was further reduced by parallel computing. A method of combining the features of the third, fourth, and fifth layers of VGG16 to enrich the features of the small targets is proposed and solves the problem of the inability of the Faster R-CNN to detect small targets. In order to improve the classification ability of the Faster R-CNN, secondary regions of interest are integrated into the classification information. The experimental results show that the detection accuracy of prohibitory, mandatory, and danger traffic signs were 99.53%, 98.40%, and 98.44%, respectively. The overall detection accuracy of the three types of traffic signs is 99.01%. Compared with similar methods, the detection precision has eclipsed previous state-of-the-art methods. The processing speed of the whole algorithm is close to 9.3 frames per second on a regular laptop, which meets the needs of real-time applications.

A faster R-CNN anchor-based strategy greatly reduces the number of detections compared with the sliding window and selective search methods, but the anchor fixed selection means the target deviates too much from the anchor point and is vulnerable to missed detection due to the influence of the bounding box regression ability. Therefore, investigating a way to add more effective a prior information to the anchor selection will be a meaningful future research direction. In addition, the fixed scales and aspect ratios of the anchor points mean that most targets lose the possibility of being overlapped with anchor areas and the original detected targets will not be detected because of the filtering of the IoU threshold. Therefore, further research should also be conducted to optimize the detection metric methods.

## Figures and Tables

**Table 1:** The comparison of different detection metrics for the grayscale + maximally stable extremal regions (MSERs), and simplified Gabor wavelets (SGW) Map + MSERs approaches.

		CTSD	GTSDB	GTSDB + CTSD
Grayscale + MSERs	Average number of proposals	321	388	343
	ROI with MSERs	99	118	105
	FNs, Recall	10, 98.12%	8, 97.1%	18, 97.76%
	Time (ms/image)	38	40	38.8
SGW Map + MSERs	Average number of proposals	178	276	211
	ROI with MSERs	56	83	65
	FNs, Recall	2, 99.62%	1, 99.63%	3, 99.63%
	Time (ms/image)	41	46	43

**Table 2:** The comparison of detection results with different methods.

Method	Anchor Scale (Pixels)	mAP%			ROI	Feature Layers	HP-RPN
		Small	Medium	Large			
Faster R-CNN	{128^2^ 256^2^ 512^2^}	12.11%	15.19%	34.12%	ROI	Conv_5	No
	{64^2^ 128^2^ 256^2^}	15.80%	16.25%	37.57%	ROI	Conv_5	No
	{16^2^ 64^2^ 128^2^}	16.53%	28.31%	38.63%	ROI	Conv_5	No
Our Approach	{128^2^ 256^2^ 512^2^}	37.53%	48.22%	59.35%	ROI	Conv_3_4_5	Yes
	{64^2^ 128^2^ 256^2^}	43.15%	51.47%	60.56%	ROI	Conv_3_4_5	Yes
	{16^2^ 64^2^ 128^2^}	60.55%	62.17%	62.56%	ROI	Conv_3_4_5	Yes
	{16^2^ 64^2^ 128^2^}	66.55%	67.17%	69.56%	SROI + ROI	Conv_3_4_5	Yes

**Table 3:** The computational times comparison of traditional Gabor wavelet (TGW), Canny and SGW [45].

Method	Number of Multiplications	Number of Addition
TGW	$32N^{2}\log_{2}N^{2}+32N^{2}$	$48N^{2}\log_{2}N^{2}+16N^{2}$
Canny	$17N^{2}$	$40N^{2}$
SGW	$16N^{2}$	$18N^{2}$

**Table 4:** The comparison of the detection accuracy with or without the information of secondary regions of interest (SROIs) and multi-layer deep convolution features.

Database	Small	Medium	Large	Total	Region	Feature Layer
GTSDB	58.26%	70.37%	84.21%	66.67%	ROI	Conv_5
CTSD	54.74%	71.66%	83.16%	72.74%	ROI	Conv_5
GTSDB	61.05%	80.16%	93.68%	81.58%	ROI+SROI	Conv_5
CTSD	64.57%	78.70%	94.73%	74.36%	ROI+SROI	Conv_5
GTSDB	89.76%	89.81%	86.84%	86.81%	ROI	Conv_3_4_5
CTSD	86.32%	86.64%	88.42%	86.27%	ROI	Conv_3_4_5
GTSDB	96.85%	100%	100%	98.53%	SROI + ROI	Conv_3_4_5
CTSD	93.68%	99.60%	100%	98.68%	SROI + ROI	Conv_3_4_5

**Table 5:** The comparison of the precision of the proposed approach with other representative approaches using the German traffic sign detection benchmark (GTSDB) and Chinese traffic sign dataset (CTSD).

Method	Database	Prohibitory	Mandatory	Danger	Total	Time(s)
Reference [53]	GTSDB	99.63%	91.33%	96.08%	97.32%	-
Reference [34]	CTSD	97.38%	95.57%	98.16%	97.10%	0.162
Reference [54]	GTSDB	98.88%	74.6%	67.3%	87.23%	-
Reference [52]	GTSDB	96%	100%	100%	97.44%	0.130
Our Method	GTSDB	98.76%	97.96%	98.41%	98.53%	0.111
Our Method	CTSD	99.24%	97.84%	98.45%	98.68%	0.106
Our Method	GTSDB + CTSD	99.53%	98.40%	98.44%	99.01%	0.108
